# Antinociceptive and Anti-Inflammatory Effects of Orally Administrated Denatured Naja Naja Atra Venom on Murine Rheumatoid Arthritis Models

**DOI:** 10.1155/2013/616241

**Published:** 2013-03-24

**Authors:** Kou-Zhu Zhu, Yan-Li Liu, Jin-Hua Gu, Zheng-Hong Qin

**Affiliations:** ^1^Department of Pharmacology and Laboratory of Aging and Nervous Diseases, Soochow University School of Pharmaceutical Sciences, 199 Ren Ai Road, Suzhou 215123, China; ^2^Department of Pharmacy, Wuxi People's Hospital Affiliated to Nanjing Medical University, Wuxi 214073, China; ^3^Department of Pharmacology, Nantong University School of Medicine, Nantong 226001, China

## Abstract

To investigate the antinociceptive and anti-inflammatory activities of the denatured Naja Naja atra venom (NNAV) in rheumatoid arthritis-associated models, the denatured NNAV (heat treated; 30, 90, 270 **μ**g/kg), the native NNAV (untreated with heat; 90 **μ**g/kg), and Tripterygium wilfordii polyglycoside (TWP, 15 mg/kg) were administrated orally either prophylactically or therapeutically. We measured time of licking the affected paw in formaldehyde-induced inflammatory model, paw volume in egg-white-induced inflammation, and granuloma weight in formalin-soaked filter paper-induced granuloma. For adjuvant-induced arthritis (AIA) rats, paw edema, mechanical withdrawal threshold, serum levels of TNF-**α** and IL-10, and histopathological changes of the affected paw were assessed. We found that the denatured NNAV (90, 270 **μ**g/kg) significantly reduced time of licking paw, paw volume, and granuloma weight in above inflammatory models and also attenuated paw edema, mechanical hyperalgesia, and histopathology changes in AIA rats. Additionally, the increase in serum TNF-**α** and the decrease in serum IL-10 in AIA rats were reversed by the denatured NNAV. Although the native NNAV and TWP rendered the similar pharmacological actions on the above four models with less potency than that of the denatured NNAV, these findings demonstrate that oral administration of the denatured NNAV produces antinociceptive and anti-inflammatory activities on rheumatoid arthritis.

## 1. Introduction

Rheumatoid arthritis (RA) is a systemic and progressive autoimmune disease, which affects about 0.5–1% of the population worldwide. The disease is characterized by pain, swelling, and stiffness of multiple joints. Although the pathogenesis of RA remains obscure, many pathogenic pathways that are involved in the development of RA have been revealed over the past years. A generally accepted viewis the disorder of the immune system. B lymphocytes may play a predominant role in the pathogenesis of RA. For the treatment of rheumatoid arthritis, strategies have shifted from controlling symptoms (such as nonsteroidal anti-inflammatory drugs and corticosteroids) to restraint the disease process with the suppression of immune system (the usage of disease-modifying antirheumatic drugs (DMARDs) and biologic agents) [1]. However, the current therapies for RA are often unsatisfactory, because of inadequate efficacy or various side effects, including gastrointestinal disorders, immunodeficiency, and humoral disturbances. Therefore, it is urgent to find candidates for the development of new drugs in RA. 

Venom therapy, as complementary and alternative medicine approach, has been used for thousands of years to treat arthritis in folk medicine. Some biotoxins, such as bee venom, have been reported to have antiarthritis and pain-relieving activity in a number of literatures [2]. In addition, antiarthritis effects of snake venoms have also been reported recently. Crotalus durissus terrificus venom, which contains crotoxin as a major active component, significantly inhibited edema and migration of polymorphonuclear cells in carrageenan-induced arthritis in mice [3, 4]. Similarly, the anti-arthritic activity of Indian monocellate cobra venom was also verified [5]. We have previously reported that cobratoxin (CTX), the long-chain *α*-neurotoxin from Thailand cobra venom, had anti-inflammatory and antinociceptive effects on adjuvant arthritis [6] and can relieve the formalin-induced inflammatory pain in rats [7]. Naja Naja atra (Chinese cobra) and itstoxic components were considered as a medicine in traditionalChinese medicine. However, though there are many reports that show that the crude venom or components from Naja Naja atra have analgesic effects [8–12], to our knowledge, there is no report on the pharmacological actions of oral administered denatured Naja Naja atra venom (NNAV) in rheumatoid arthritis model. 

Oral administration of drug is the most common and convenient way, but it is usually assumed that the oral administration of peptides will be biologically ineffective due to enzymatic digestion or chemical degradation in gastrointestinal tract. However, contrary to the general idea, Giorgi et al. [13] reported that a low molecular weight component from Crotalus durissus terrificus venom induced analgesia when given orally. Similarly, oral administration of neurotoxin from king cobra venom produced the analgesic action [10]. Recently, the absorption of ^125^I-labeled neurotoxin from rectum was also confirmed in rabbits [14]. Therefore, we speculated that oral administration of NNAV might produce the pharmacological effects on AIA rats. 

In view of the toxicity of snake venom, there are several methods used to reduce toxicity. Zhang et al. [15] reported that oxidative modification of the NNAV could weaken the toxicity and significantly inhibit the adjuvant-induced edema. In addition, heat treatment is the alternative way. For instance, after heating the Naja kaouthia venom at 100°C for 10 min, the LD_50_ of the denatured Naja kaouthia venom increased 14% compared to the native one [16]. In accordance with the report, in the preliminary study we found that LD_50_ of the denatured NNAV was 20% higher than that of native NNAV (about 100 mg/kg for native NNAV and 120 mg/kg for denatured NNAV). 

Therefore, in this study we applied the oral administration of denatured NNAV to investigate whether it bears anti-inflammatoryand antinociceptive effects on murine rheumatoid arthritis-associated models. 

## 2. Materials and Methods

### 2.1. Animals

48 Kunming mice (half male and half female, weighing 18–20 g) and 232 male Sprague-Dawley rats (weighing 180–220 g) were purchased from the Center for Experimental Animals of Soochow University. Animals were fed ad libitum and housed in individual cages with a controlled ambient temperature (22 ± 2°C), humidity (40–70%), and a 12 h light/dark cycle. Animals were acclimated to the housing conditions and handled for 3-4 days before experiments. All experiments were performed between 09:00 AM and 5:00 PM. All experimental procedures were conducted in accordance with the NIH Guidelines for the Care and Use of Laboratory Animals (NIH Publications number 80-23, revised 1996). The study protocol was approved for the use of animals in research by the local ethics committee. 

### 2.2. Reagents and Drug Administration

Naja Naja atra venom, purchased from Rainbow snake farm (Yujiang, Jiangxi Province, China), was dissolved in sterile water. NNAV was heated in boiling water for 10 minutes and then was left to cool naturally (NNAV+). Unheated NNAV was also dissolved in sterile water without heating (NNAV−). Both NNAV+ and NNAV− were stored at 4°C until use. 

Because Tripterygium wilfordii polyglycoside (TWP) has been widely used in the treatment of rheumatoid arthritis, we chose it as positive drug. TWP was purchased from MeitongPharmaceutical Co, Ltd. (Jiangsu Province, China). Complete Freund's adjuvant (CFA) was purchased from Sigma (Saint Louis, MO, USA) and suspended in a 1 : 1 oil/saline emulsion. 

The denatured NNAV (30, 90, 270 *μ*g/kg), the native NNAV (90 *μ*g/kg), and TWP (15 mg/kg) were administered orally. The reasonable doses of the NNAV and TWP were utilized according to the literature [4, 5], the LD_50_ of the denatured NNAV and preliminary test results (data not shown). Prophylactical or therapeutical administration depended on the experimental protocol in different animal models. 

### 2.3. Analysis of the Denatured and Native NNAV Components

Denatured NNAV and native NNAV were analyzed by loading equivalent amounts of total NNAV (4–16 *μ*g) onto 15% SDS-PAGE gel. After incubating gel with Coomassie brilliant blue staining R 250 for 2 h, gel was eluted with bleaching solution (H_2_O: 100% alcohol: 36% acetic acid = 3 : 1 : 1, volume ratio) overnight. The proteins signal was read with an Odyssey Western Blot Analysis system (LI-COR Biosciences). 

### 2.4. Formaldehyde-Induced Inflammatory Pain and NNAV Pretreatment

KM mice were administrated with the denatured NNAV (30, 90, 270 *μ*g/kg, i.g.) or the native NNAV (90 *μ*g/kg, i.g.) or saline, followed by subcutaneous injection of 20 *μ*L 5% formaldehyde into the right hind paw 6 h later [17]. After the formalin injection, time spent in licking the injected paw, divided into phase 1 (0–5 min) and phase 2 (20–30 min), was recorded by stopwatch. 

### 2.5. Egg-White-Induced Nonspecific Inflammation Model

Rats were given orally with the denatured NNAV or the native NNAV or TWP (15 mg/kg) once daily for 5 days before injection of 0.1 mL of 10% (v/v) fresh egg white, dissolved in normal saline, into the right hind paws [18]. Paw volume was measured at 0, 0.5, 1, 2, and 4 h after injection of egg white by using the method of water displacement (YLS-7C, YiyanTechnology Co, Ltd, Jinan, Shandong Province, China). Change in paw volumebefore and afterinjection ofegg white was calculated. 

### 2.6. Formalin-Soaked Filter Papers-Induced Granuloma

Granulomas in rats were induced by 7% formalin-soaked filter papers [19]. Filter paper pallets, 6 mmin diameter and 0.36 mm thick, were soaked in 7% formaldehyde solution. Pallets were placed in the subcutaneous tissues at each axillary area under sterile conditions. After surgery, rats were treated orally with NNAV, TWP, or saline once daily for 7 days. At the 7th day, all rats were killed and granuloma was dissected. The fresh granuloma was weighed as wet weight. After drying for 12 h at 60°C, dry weight was recorded. 

### 2.7. Adjuvant-Induced Arthritis (AIA)

AIA in rats was induced by an intra-articular injection of 100 *μ*L CFA into knee joint [5]. As a control, 100 *μ*L of saline was injected. The effects of drugs on AIA rats were tested using the following protocols: (1) pretreatment protocol, rats were administrated with the denatured NNAV, the native NNAV, or TWP once daily for 5 days before injection of CFA; (2) posttreatment protocol, rats were given orally with these drugs from the 11th day to 28th day after injection of CFA till the end of experiment. 

### 2.8. Measurement of the Ankle Joint Circumference and Volume of the Right Hind Paw in AIA Rats

The ankle joint circumference was measured using a flexible tape. Volume of the right hindpaw was determined with the method of water displacement. All rats were marked with a red marker pen in the ankle joints. Eachmeasurementwasperformed by vertical insert of hindpawinto themeasuringcupto reachthered line. Before injection of CFA, the volume of right hindpaw was determined as the baseline. At different time points after injection of CFA, the volume of the right hindpaw was determined again. 

Paw volume and ankle joint circumference were determined at 0 (time before injection of adjuvant or saline), 6, 24, and 72 h after adjuvant injection for preventive protocol and 0 (time prior to the injection of CFA), the 10th, 12th, 20th, and 28th day after adjuvant injection for treatment protocol. Change in paw volume and ankle joint circumference before and after CFA injection was calculated. 

### 2.9. Assessment of Mechanical Pain Response in AIA Rats

Rats were placed in a plastic cage with a wire mesh bottom, which allowed full access to the paws. Behavioral accommodation was allowed for about 15 min, until exploratory behavior and major grooming activities ceased. The area tested was the midplantar of the right hindpaw. The paw was touched with 1 of a series of 10 von Frey hairs (1, 1.4, 2, 4, 6, 8, 10, 15, 26, and 60 g). Mechanical sensitivity of the plantar surface of the right hindpaw was tested in rats using the up-down method [20]. The von Frey hair (North Coast Medical Inc., Morgan Hill, CA, USA) was presented perpendicularly to the plantar surface with sufficient force to cause slight buckling against the paw and was held for approximately 6–8 s. Each test was presented at intervals of several seconds allowing for apparent resolution of any behavioral responses to previous stimuli. A positive response was counted if the paw was sharply withdrawn. Flinching immediately upon the removal of the hair was also considered as a positive response. Ambulation was considered an ambiguous response, and in such cases the stimulus was repeated. The time point of measuring mechanical pain was the same to paw volume and ankle joint circumference. 

### 2.10. Determination of Serum Levels of TNF-*α* and IL-10 in AIA Rats

Rats were anesthetized with 4% chloral hydrate (1 mL/100 g, i.p.) at the end of treatment protocol, and blood was collected from abdominal aorta. After standing for 30 min, blood was centrifuged at 3000 rpm for 10 min. Supernatant was collected and kept at −20°C for further analysis within less than 3 days. The levels of TNF-*α* and IL-10 were determined with the commercially available enzyme immunoassay kits (Yifeng Biotechnology Ltd Co. Shanghai, China). 

### 2.11. Histological Assessment of AIA Rats

Right ankle joints were separated from the right hind feet and fixed in 10% PBS-buffered formalin for 24 h, followed by decalcification in 10% EDTA. Then the joints were dehydrated and processed, and paraffin (56–58°C) blocks were prepared. Sections (5 *μ*m) were prepared, stained (hematoxylin & eosin), and examined with an optical microscopy. 

### 2.12. Statistical Analysis

All data were presented as mean ± SD. The significant differences between experimental groups and control groups were analyzed using ANOVA. Analysis of variance for repeated measurement was used where applicable. The post hoc test was Student's Newman Keuls test for quantitative values. Kruskal-Wallis *H* test was used for qualitative values, followed by the Mann-Whitney *U* test. Significance was set at *P* < 0.05. Calculations were performed using the SPSS 16.0 statistical package. 

## 3. Results

### 3.1. The Composition Changes of the Denatured NNAV

**Figure 1:**
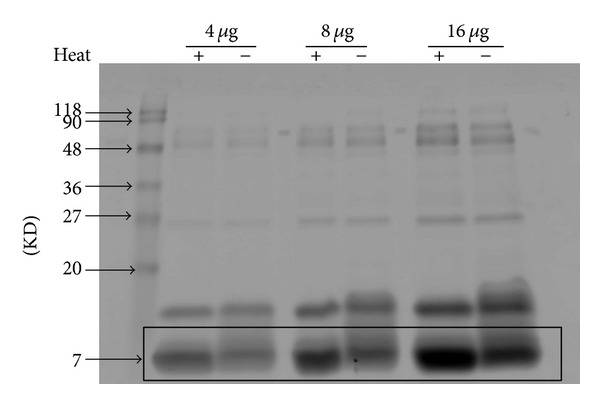
Analysis protein composition of NNAV with SDS-PAGE. NNAV was dissolved in sterile water and heated to 100°C for 10 min. Denatured or native NNAV was loaded onto 15% PAGEL and subjected to electrophoresis. The gel was stained with Coomassie brilliant blue and photographed. Noted an increase in abundance of proteins with molecular mass of 7** **KD.

As shown in Figure 1, the major components of NNAV were migrated with a molecular weight (MW) between 7 and 20 KD. Heat treatment (NNAV+) induced an increase in protein contents with about MW 7 KD. According to the literature, the protein band of MW 7 KD contained cardiotoxin and neurotoxin [21, 22]. In contrast, heat treatment did not markedly alter the components of high MW proteins (more than 20 KD) in NNAV. 

### 3.2. Inhibition of Formaldehyde-Induced Pain Response by NNAV

**Figure 2:**
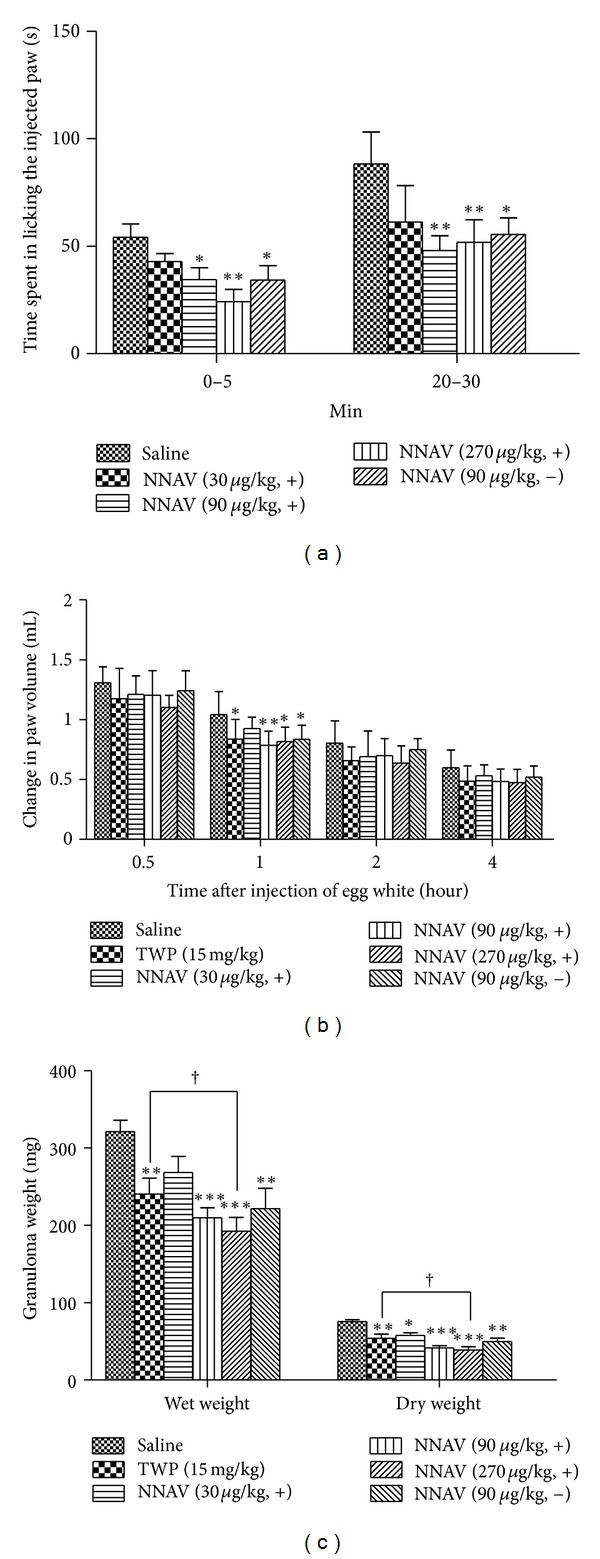
Effects of the denatured NNAV on three inflammatory models induced by the injection of formaldehyde and egg white implantation of formaldehyde-soaked filter papers. KM mice were orally administrated with the denatured NNAV (30, 90, 270 *μ*g/kg), the native NNAV (90 *μ*g/kg), or saline once every 6 h before the injection of formaldehyde into the right hindpaw (a). Time spent in licking the injected paw during phase 1 (0–5 min) and phase 2 (20–30 min) was recorded. Rats were treated with the above drugs once daily for 5 days before injection of egg white (b). Paw edema was determined by measuring the paw volume using the method of water displacement. Rats were treated with the above drugs once daily for 7 days after implantation of paper pellets (c). Data represent mean ± SD ((a) *n* = 12, (b) *n* = 10, and (c) *n* = 10). *P < 0.05, **P < 0.01, ***P < 0.001 compared with saline group; ^†^ *P* < 0.05, denatured NNAV (270 *μ*g/kg) versus TWP (15 mg/kg).

Formaldehyde-evoked biphasic nociceptive responses include an early, short-lasting response (phase 1) and a late, prolonged response (phase 2). As shown in Figure 2(a), pretreatment with the denatured NNAV (30, 90, and 270 *μ*g/kg) exhibited a dose-dependent analgesic effect during the phase 1 (0–5 min) response. Time spent in licking the injected paw in phase 1 was inhibited by 30% (90 *μ*g/kg) and 45% (270 *μ*g/kg). In the phase 2 (20–30 min), the denatured NNAV reduced the licking time by 43% (90 *μ*g/kg) and 38% (270 *μ*g/kg). Similarly, the native NNAV (90 *μ*g/kg) also exhibited the analgesic effect in the both phases. However, we failed to find the significant difference in licking time between the denatured and native NNAV (90 *μ*g/kg). 

### 3.3. Inhibition of Egg-White-Induced Edema by NNAV

As shown in Figure 2(b), injection of 100 *μ*L 10% fresh egg white noticeably elicited induced acute inflammatory model and the inflammatory symptom-paw edema peak at 0.5–1 h after injection then gradually relieved but persisted for 4 h [23]. Pretreatment with the denatured NNAV (90, 270 *μ*g/kg), native NNAV(90 *μ*g/kg), and TWP (15 mg/kg) all significantly reduced paw edema at 1 h after injection of egg white, when compared to the saline group. However, significant difference could not be found between the denatured and native NNAV (90 *μ*g/kg). 

### 3.4. Inhibition of Formalin-Soaked Filter Papers-Induced Granuloma Formation by NNAV

After implanting formaldehyde-soaked filter paper, rats were treated with the denatured NNAV, TWP, and native NNAV once daily. At the 7th day (the most severe period of granuloma formation), rats were killed, granuloma was dissected, and the weight of the granuloma was measured. The denatured NNAV exhibited a dose-dependent anti-inflammatory action. Granuloma in wet weight was inhibited by 37% (90 *μ*g/kg) and 42% (270 *μ*g/kg). In dry-weight, significantinhibitionin various dosesof the denatured NNAV, native NNAV or TWP was also observed, as is shown in Figure 2(c). There is a significant difference in either wet weight or dry weight between TWP and denatured NNAV 270 *μ*g/kg, indicating that denatured NNAV was more effective. 

### 3.5. Effects of NNAV on the Inflammatory Edema in AIA Rats

The injection of complete Freund's adjuvant (CFA) induced primary inflammatory signs and hyperalgesia at the inoculation site of hindpaw within hours after injection. Subsequently, secondary inflammation and nociceptive symptoms appeared between the 10th day and 28th day after inoculation. For investigation of the antinociceptive and anti-inflammatory effects of NNAV in different inflammatory phases, we used prophylactic and therapeutic treatment protocols. 

### 3.6. Paw Volume

**Figure 3:**
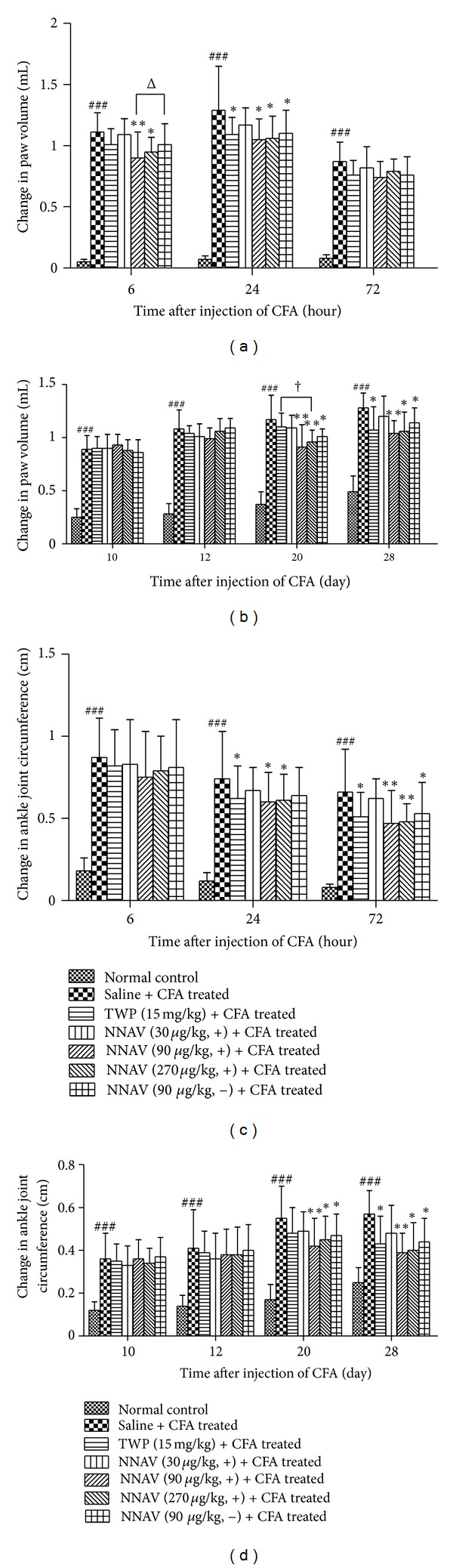
Inhibition of edema by the denatured NNAV in AIA rats. AIA rats were orally administrated once daily for 5 days before injection of CFA ((a), (c)) or from the 11th day to the 28th day after injection of CFA ((b), (d)) with the denatured NNAV (30, 90, 270 *μ*g/kg), the native NNAV (90 *μ*g/kg), or TWP (15 mg/kg). The volume of right hindpaw was measured using the method of water displacement. Changes in the circumference of ankle joint were determined with a flexible tape. ((a), (c)) and ((b), (d)) represented pretreatment protocol and posttreatment protocol, respectively. Data represent mean ± SD (*n* = 8). ^###^ *P* < 0.001 compared with normal control group, *P < 0.05, **P < 0.01 compared with untreated arthritis group; ^†^ *P* < 0.05 denatured NNAV (270 *μ*g/kg) versus TWP (15 mg/kg); ^Δ^ *P* < 0.05 denatured NNAV (90 *μ*g/kg) versus native NNAV (90 *μ*g/kg).

As shown in Figure 3(a), pretreatment with the denatured NNAV (90, 270 *μ*g/kg, i.g.) for 5 days before the injection of CFA significantly reduced ipsilateral paw edema at 6 and 24 h, when compared to the saline group. In contrast, the native NNAV (90 *μ*g/kg) and TWP (15 mg/kg) only alleviated paw edema at 24 h after injection of CFA. It is interesting that the denatured NNAV was more effective than the native NNAV with the same dose of 90 *μ*g/kg at 6 h. In the therapeutic protocol (Figure 3(b)), the denatured NNAV was administrated orally once daily from the 11th day to 28th day after injection of CFA. Although there was no difference in paw volume among different groups of AIA rats at the 12th day, the edema was significantly alleviated by the denatured NNAV (90, 270 *μ*g/kg) and native NNAV (90 *μ*g/kg) at the 20th and 28th day. However, we failed to find the significant difference between the denatured and native NNAV (90 *μ*g/kg). Different from NNAV, TWP did not produce the anti-inflammatory effect at the 20th day but reduced paw edema at the 28th day. The relatively low dosage of TWP maybe accounts for the discrepancy. Indeed, the results were consistent with the previous report, in which TWP, fed (10 mg/kg/d) from 17th to 25th after injection of CFA, also did not produce significant protecive effect [24]. 

### 3.7. Ankle Joint Circumference

In addition to paw volume, we also used ankle joint circumference as the indicator of inflammatory edema to evaluate the anti-inflammatory effect of NNAV on AIA rats. For prophylactical treatment (Figure 3(c)), the denatured NNAV significantly reduced adjuvant-induced enlargement of ankle joint at 24 h and 72 h, when compared to the saline group. TWP had similar effects as denatured NNAV on ankle joint edema. However, the native NNAV only had similar effect as the denatured NNAV at 72 h. In the therapeutic experiments (Figure 3(d)), there was no difference in the circumference of ankle joint among different groups of arthritis rats at the 12th day, but ankle joint circumference significantly decreased by the denatured NNAV (90, 270 *μ*g/kg) and native NNAV (90 *μ*g/kg) at the 20th and 28th day. Consistent with the observation of paw volume, TWP only reduced ankle joint circumference at 28th day but not at 20th day.

### 3.8. Effects of NNAV on Hyperalgesia in AIA Rats

**Figure 4:**
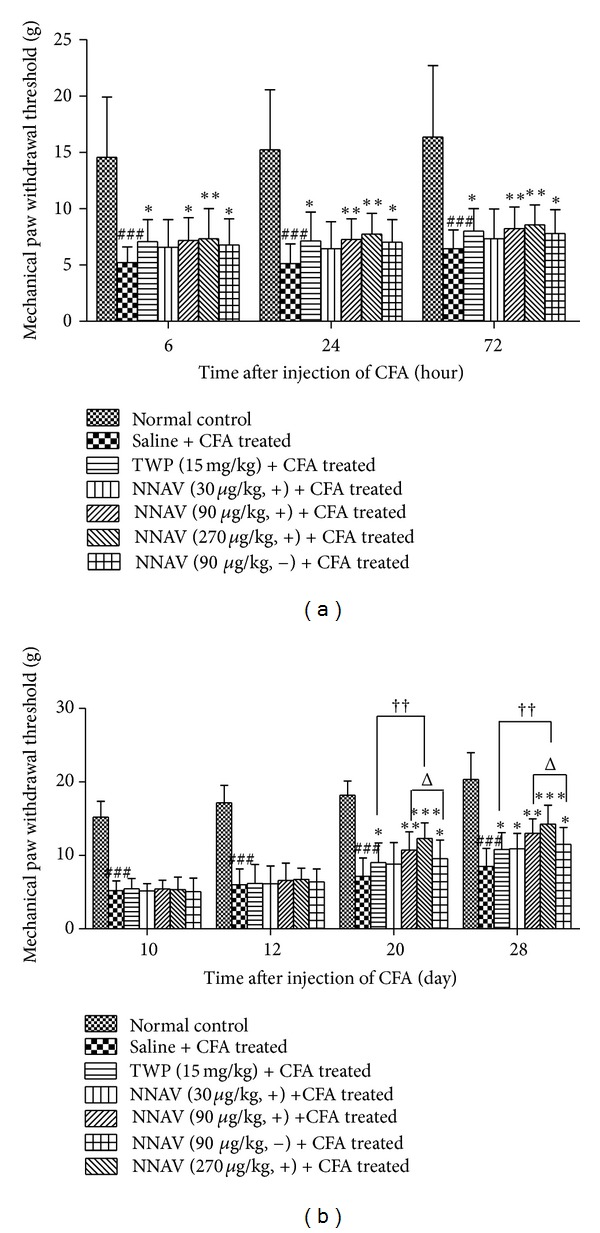
Effects of the denatured NNAV on hyperalgesia in AIA rats. Mechanical allodynia was measured by the up-down method. (a) and (b) represent pretreatment protocol and posttreatment protocol, respectively. Data represent mean ± SD (*n* = 8). ^###^ *P* < 0.001 compared with normal control group, *P < 0.05, **P < 0.01, ***P < 0.001 compared with untreated arthritis group, ^††^ *P* < 0.05 denatured NNAV (270 *μ*g/kg) versus TWP (15 mg/kg), ^Δ^ *P* < 0.05 denatured NNAV (90 *μ*g/kg) versus native NNAV (90 *μ*g/kg).

As shown in Figure 4, CFA injection into paw induced marked mechanical hyperalgesia, as evidenced by reduced nociceptive thresholds not only during the primary inflammatory period (within 72 hours), but also during the second inflammatory period (from 10th day to 28th day). In the prophylactic treatment protocol (Figure 4(a)), the denatured NNAV (90, 270 *μ*g/kg), the native NNAV (90 *μ*g/kg), and TWP all significantly inhibited CFA-induced mechanical hyperalgesia, we could not observe the statistical difference between the denatured NNAV and native NNAV or TWP. 

In the therapeutic protocol (Figure 4(b)), although the nociceptive threshold did not change in all groups of AIA rats at the 12th day, NNAV including the denatured NNAV (90, 270 *μ*g/kg), the native NNAV (90 *μ*g/kg) and TWP all could significantly alleviate the mechanical hyperalgesia at the 20th day and the 28th day. It was noticeable that the denatured NNAV was more effective than the native NNAV with the dose of 90 *μ*g/kg at the 20th day (12.12% increase of nociceptive threshold) and at the 28th day (12.95% increase of nociceptive threshold). Additionally, we also found that the denatured NNAV (270 *μ*g/kg) group had 36.32% or 41.56% more higher nociceptive threshold than TWP (15 mg/kg) group at the 20th or the 28th day. 

### 3.9. Effects of NNAV on Serum Cytokine Levels in AIA Rats

**Figure 5:**
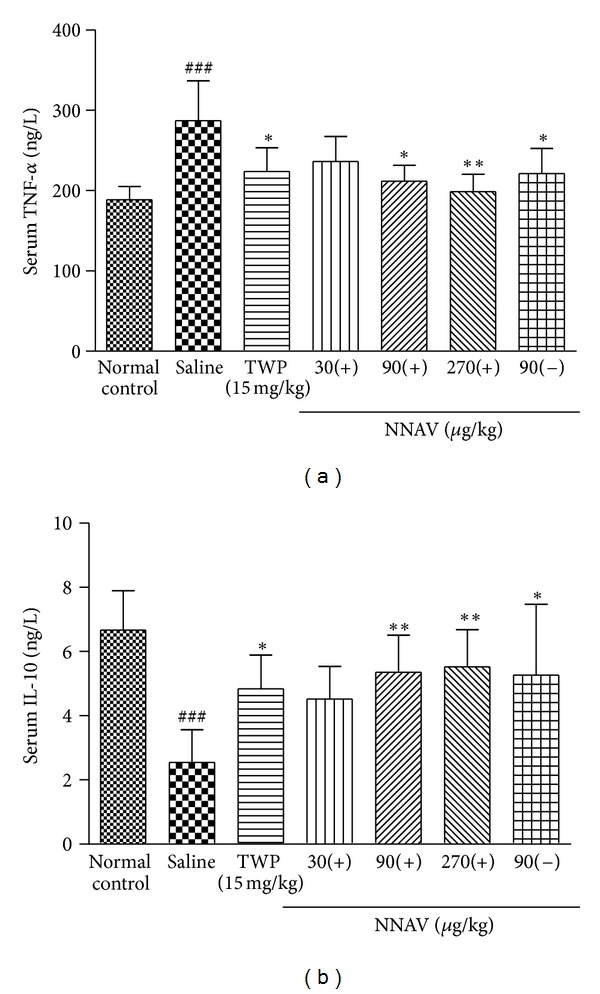
Effects of NNAV on serum cytokine levels in AIA rats. Blood serum was collected on the day of 28th after CFA. Serum levels of TNF-*α* and IL-10 were measured with the enzyme immunoassay kits. Values are the mean ± SD of 6 rats per group. ^###^ *P* < 0.001 compared with normal control group, *P < 0.05, **P < 0.01 compared with untreated arthritis group.

There was a significant increase in the concentrations of the proinflammatory cytokine TNF-*α* in the serum of AIA rats on the 28th day after CFA injection. Posttreatment with the denatured NNAV (90, 270 *μ*g/kg), the native NNAV (90 *μ*g/kg), and TWP, starting on the 11th and ending on the 28th day after CFA, had a significant inhibitory effect on the levels of TNF-*α* (Figure 5(a)). However, the concentration of anti-inflammatory cytokine IL-10 decreased in rats after CFA treatment. Posttreatment with the denatured NNAV (90, 270 *μ*g/kg), the native NNAV (90 *μ*g/kg), and TWP all could reverse the CFA-induced decline in IL-10 (Figure 5(b)). 

### 3.10. A and Synovium in AIA Rats

**Figure 6:**
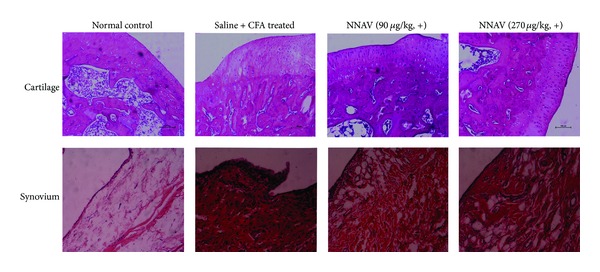
Histological examination of ankle joints. AIA rats were treated therapeutically with the denatured NNAV (30, 90, 270 *μ*g/kg, i.g.). Sections were stained with hematoxylin and eosin (cartilage 100x, synovium 200x). Compared with the untreated AIA joint, infiltration of inflammatory cells and accumulation of collagen and hyperplasia of articular cartridge were markedly ameliorated by posttreatment with denatured NNAV.

As shown in Figure 6, articular cartilage in saline-treated AIA rats had severe hyperplasia, compared with normal control rats of which the articular cartilage showed smooth surfaces with intact layers of flattened cells. Furthermore, the synovium was histopathologically analyzed with HE staining. The results showed synovial lining layer hypertrophy, subliming infiltration with mononuclear cells, and pannus formation. These abnormalities of articular cartilage and synovium were significantly alleviated in AA rats after the administration of the denatured NNAV (90, 270 *μ*g/kg) once daily from the 11th to the 28th day after injection of CFA. In addition, the native NNAV (90 *μ*g/kg) and TWP also showed similar protective effects on the histopathological abnormalities (data not shown). 

## 4. Discussion

In the present study, the main finding is that oral administration of the denatured NNAV produced strong anti-inflammatory and analgesic effects. Several animal models were applied to investigate the pharmacological effects of NNAV on inflammation. One was formalin-induced inflammatory pain model, which is a valid and reliable model of inflammation-mediated nociception. Intradermal injection of formalin into the mice paw induces two phases of nociceptive response evidenced by flinching, licking, or biting of the affected paw. Using this model, we found that after pretreatment with the denatured NNAV (90, 270 *μ*g/kg), time spent in licking the injected paw was markedly inhibited in the two phases, indicating its analgesic effects. 

The second animal model was egg-white-induced nonspecific inflammation, which is fit for evaluation of anti-inflammatory agents and is used frequently to assess the antiedematous effect of natural products [25]. Injection of egg white caused the foot edema, redness, and the increase in skin temperature of the affected paw. In this study, we found that the denatured NNAV effectively inhibited the nonspecific inflammation. The evidence is that pretreatment of the denatured NNAV (90, 270 *μ*g/kg) significantly reduced paw edema 1 h after injection of egg white. 

The third model was formalin-soaked filter papers-induced granuloma, which has been a standardized quantitative anti-inflammatory assay [26]. In rats, the growth of the granulation tissue around the paper was markedly depressed when the denatured NNAV was administrated orally during the period after subcutaneous implantation of formalin-soaked filter papers. The results suggest that the denatured NNAV is effective in the proliferation stage of inflammation. 

The final model is adjuvant-induced arthritis (AIA) in rats. Due to the close resemblance to human rheumatoid arthritis in terms of clinical symptoms, histological and immunological features, it is a well-established model that has been used to investigate the pathogenesis of rheumatoid arthritis and evaluate the analgesic potential or anti-inflammatory effects of drugs. Injection of complete Freund's adjuvant into the paw induces acute inflammatory responses and hyperalgesia, such as protecting the affected paw and avoiding putting body weight on the paw at the site of inoculation within hours. Subsequently, chronic inflammation and pronociceptive symptoms appear between the 10th and 30th day after inoculation. 

In AIA rats, the efficacy of the denatured NNAV was investigated when administered prophylactically as well as therapeutically. We found that the oral administration of the denatured NNAV significantly attenuated CFA-induced paw edema and mechanical hyperalgesia not only during the primary inflammatory period (within 72 hours), but also during the second inflammatory period (from the 10th day to the 28th day). In addition to examining the paw edema and nociceptive thresholds, we evaluated the pharmacological effect on histological changes in AIA rats. The results demonstrated that abnormalities of articular cartilage and synovium were significantly alleviated. 

To investigate the possible anti-inflammatory mechanisms, we also measured the serum concentrations of TNF-*α* and IL-10 on the 28th day after CFA injection. TNF-*α* plays a critical role in the pathological change in the process of RA [27]. In the process of bone erosion, TNF-*α* triggers the production of other cytokines and endothelial adhesion molecules, stimulates collagenase, and induces osteoclast differentiation [28]. Furthermore, TNF-*α* exerts its arthritogenic potency through the induction of IL-1. Therefore, TNF-*α* has been shown to be the dominant player in the induction of inflammation and bone erosion in RA [29, 30]. Williams et al. found that anti-mouse TNF-*α* monoclonal antibody administered after disease onset ameliorated both inflammation and the joint damage in the collagen type II model of arthritis [31]. At the present, the TNF-*α* inhibitors remain as the most effective drugs fighting RA [32]. Increasing evidence suggests that IL-10 is an important anti-inflammation cytokine in RA. Posttreatment with IL-10 reduced the swelling in collagen type II-induced arthritis [33]. Our data demonstrated that CFA induced a significant elevation of serum TNF-*α*, but reduced the levels of IL-10. Denatured NNAV reduced the levels of TNF-*α* and resumed the production of IL-10, indicating that denatured NNAV has anti-inflammatory and analgesic activities by regulating the immune system. 

Although thecomposition of crude venomis complex,it can be divided into *α*-neurotoxin (including long-chain *α*-neurotoxin: “cobratoxin” and short-chain *α*-neurotoxin: “cobrotoxin”), cardiotoxin, cobra venom factor (CVF), and so forth. Our recent study has shown that cobratoxin suppressed inflammation and pain in AIA rats [6]. We also found that cobratoxin inhibited pain-evoked discharge of neurons in thalamic parafascicular nucleus in rats [34]. In addition to cobratoxin, active components from NNAV have been studied for their actions in arthritis and nociception. It has been reported that cobra venom factor (CVF) suppressed yersinia and adjuvant-induced arthritis in rats by decomplementation [35]. Cardiotoxin (Najanalgesin) was isolated from NNAV and reportedly had peripheral and spinal antihyperalgesic activity in a rat experimental model of neuropathic pain [12]. Researcheson the activities of single component from crude venom are helpful for elucidating pharmacological base and molecular mechanisms of NNAV's actions. We assume that the cobrotoxin (10%), CVF (1-2%), and cardiotoxin (25–50%) in the denatured NNAV together produce potent anti-inflammatory and analgesic actions in AIA rats. 

In this study, it is noticeable that denatured NNAV is, to some extent, better than native NNAV. Based on SDS-PAGE, we found that heat treatment increased the concentration of low molecular weight proteins about 7 KD. This molecular mass may include cardiotoxin and cobrotoxin, or some breakdown fragments from large molecular weight proteins. These changes may account for the better pharmacological effects of denatured NNAV than native NNAV. In addition, heat treatment also increased its oral LD_50_ from 102 mg/kg to 121 mg/kg, suggesting a reduction in acute toxicity (data not shown). 

Oral administration of crude venom is the most convenient and safe way. It will hold tremendous promisefortheclinical applicationofdrugs. It has been reported in Chinese literature that neurotoxin in cobra venom can be rapidly absorbed through rectal mucosa [14]. Even though we are not sure what amount of intact peptides in denatured NNAV would be absorbed in the gut, but, based on its LD_50_ dosage and produce pharmacological actions, we believe that oral denatured NNAV can get into general circulation and exerts its biological effects. We have assessed subchronic toxicity of oral administered denatured NNAV and found that continuous daily administration for 33 days at the dose 30–270 *μ*g/kg did not produce any sign of toxicity. This property renders denatured NNAV a good potential for clinical usage for inflammation related diseases. 

Tripterygium wilfordii polyglycoside (TWP) is a Chinese herb with immunosuppressive and anti-inflammatory effects and an established history of use in the treatment of rheumatoid arthritis [36, 37]. However, someclinical adverse events have reported in randomized clinical trials. The most common side effect of TWP is gastrointestinal tract disturbances, such as anorexia, diarrhea, and abdominal pain [36]. Thus safer and more effectivedrugisurgently needed. Based on results between the denatured NNAV and TWP on anti-inflammation and antinociceptive, we found that TWP was less potent than denatured NNAV in some aspects. On the other hand, different from many other anti-RA drugs, NNAV may not be an immunosuppressant as it does not have an effect on immune organs [38]. 

## 5. Conclusions

The present study demonstrated that the oral administration of denatured NNAV could attenuate the inflammation-induced pain and joint damage by regulating the immune system. Therefore, the denatured NNAV may be a novel therapeutic drug for RA.
